# A coded aperture microscope for X-ray fluorescence full-field imaging

**DOI:** 10.1107/S1600577520012308

**Published:** 2020-10-21

**Authors:** D. P. Siddons, A. J. Kuczewski, A. K. Rumaiz, R. Tappero, M. Idir, K. Nakhoda, J. Khanfri, V. Singh, E. R. Farquhar, M. Sullivan, D. Abel, D. J. Brady, X. Yuan

**Affiliations:** a National Synchrotron Light Source II, Brookhaven National Laboratory, Upton, NY 11973, USA; b Stony Brook University, Stony Brook, NY 11794, USA; c Louisiana State University, Baton Rouge, LA 70803, USA; d Case Western Reserve University, Cleveland, OH 44106, USA; e Duke University, Durham, NC 27708, USA; f Nokia Bell Labs, Murray Hill, NJ 07974, USA

**Keywords:** coded aperture, X-ray microscope, full-field, fluorescence

## Abstract

The design and construction of an instrument for full-field imaging of the X-ray fluorescence emitted by a fully illuminated sample are presented. The aim is to produce an X-ray microscope with a few micrometers spatial resolution, which does not need to scan the sample. Since the fluorescence from a spatially inhomogeneous sample may contain many fluorescence lines, the optic which will provide the magnification of the emissions must be achromatic, *i.e.* its optical properties must be energy-independent. The only optics which fulfill this requirement in the X-ray regime are mirrors and pinholes. The throughput of a simple pinhole is very low, so the concept of coded apertures is an attractive extension which improves the throughput by having many pinholes, and retains the achromatic property. Modified uniformly redundant arrays (MURAs) with 10 µm openings and 50% open area have been fabricated using gold in a lithographic technique, fabricated on a 1 µm-thick silicon nitride membrane. The gold is 25 µm thick, offering good contrast up to 20 keV. The silicon nitride is transparent down into the soft X-ray region. MURAs with various orders, from 19 up to 73, as well as their respective negative (a mask where open and closed positions are inversed compared with the original mask), have been made. Having both signs of mask will reduce near-field artifacts and make it possible to correct for any lack of contrast.

## Abstract 2

First results from a 10 µm MURA coded aperture optic for a full-field X-ray fluorescence microscope are presented.

## 1. Introduction   

Current X-ray microprobe beamlines raster-scan the sample through a focused X-ray beam, and use an energy-resolving point detector to separate the contributions of the various elements at each point on the sample. Although this method has many advantages, it suffers from the need to mechanically move the sample through the beam, which is inevitably rather slow, particularly if the requirement is for 3D tomographic imaging, since this requires two translational scans for each of many rotational positions. An instrument capable of recording an image directly eliminates two of those dimensions, and can replace the rotation with a single translation for tomography (Takeuchi *et al.* 2009 ▸, 2010 ▸). A full-field microscope should collect fluorescence from all points on the sample simultaneously and project them onto an imaging detector. To image elemental contributions we need:

(i) An achromatic imaging optical element to magnify the fluorescence signal to match the desired resolution to the detector pixel size.

(ii) A detector which collects energy spectra from each detector pixel.

In this paper we demonstrate an achromatic imaging system, the coded aperture array. The hyperspectral imaging detector is also under development at Brookhaven National Laboratory, but is the subject of another work.

## 2. Coded apertures   

### 2.1. Modified uniformly redundant arrays   

The pinhole camera is the oldest and simplest imaging optic. It suffers from the inverse relationship between light collection efficiency and spatial resolution. The coded aperture array was developed as a way to break that relationship, by using arrays of pinholes in known locations. Even though the resulting image *I*(*x*,*y*) is an overlay of many weak images, it is in principle possible to reconstitute the image mathematically. This became realistic as the era of digital computers arrived.

When the photons generated by an object *O*(*x*,*y*) propagate through the coded aperture mask of binary function *M*(*x*,*y*), the resulting image projected onto the detector is *I*(*x*,*y*),
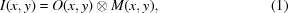
with ⊗ denoting a convolution.

**Figure 1:**
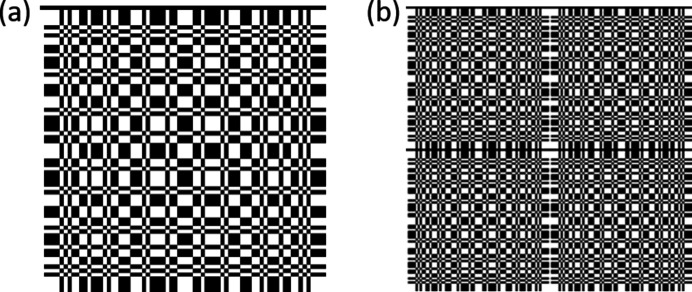
Pattern of an order 73 (*a*) single and (*b*) tiled MURA.

Various forms of coded aperture arrays have been demonstrated, with perhaps the most successful being the modified uniformly redundant array (MURA) proposed by Gottesman & Fenimore (1989 ▸). It has the advantage of having both high efficiency (50% open area) and a perfect system point spread function (SPSF). This means that the convolution of the MURA mask *M*(*x*,*y*) with its anti-mask *H*(*x*,*y*) is a perfect delta function,
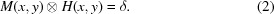
A MURA mask *M*(*x*,*y*) of order *p*, a prime number, and its anti-mask *H*(*x*,*y*) are square lattices of size *p* × *p*, with *x* and *y* varying from 0 to *p* − 1 and are defined as
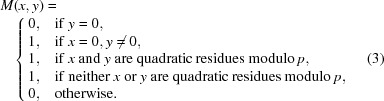


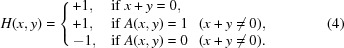
The retrieved object is then obtained from the detected image on the detector as
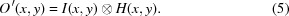
For this work, we chose to construct MURA masks with a unit pinhole of 10 µm × 10 µm square, and designed arrays of order 19, 37 and 73. For a given pinhole size, the higher the order of the mask, the bigger the field of view of the system. Fig. 1 ▸(*a*) shows the pattern for order 73. It is usual to tile such structures to increase its useful field of view. Shown in Fig. 1 ▸(*b*) is the layout of the typical 2 × 2 tiling that we sent for manufacturing.

### 2.2. Optic fabrication   

**Figure 2:**
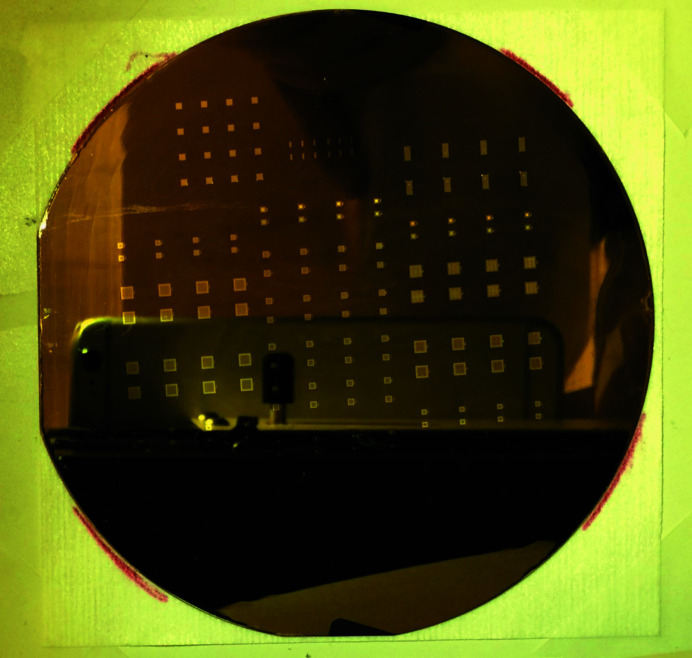
The finished silicon wafer prepared using the LIGA technique at the X-ray lithography facility at CAMD. It holds MURA of order 19, 37 and 73 as well as other objects such as single pinholes and slits.

**Figure 3:**
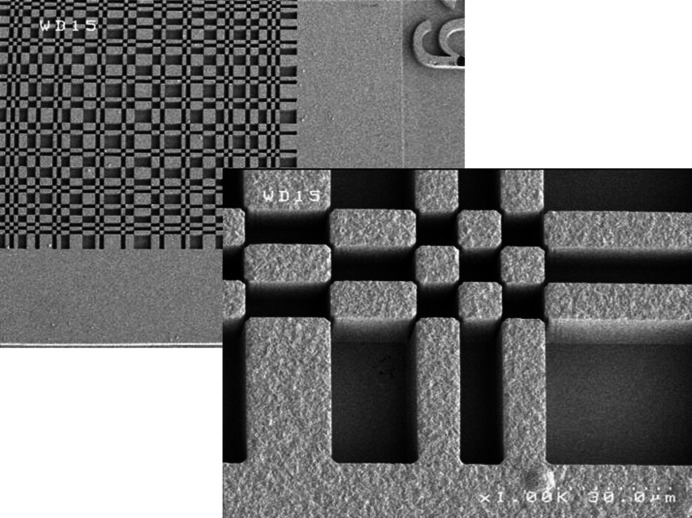
Electron micrographs of the MURA coded aperture with 10 µm holes manufactured using the LIGA technique.

Looking at the pattern of Fig. 1 ▸, it is obvious that the MURA mask is not self-supporting. Previous attempts to use such apertures have been forced to use self-supporting variants. Typically they were made by drilling or etching small holes in sheet metal such that no two holes touched (so-called NTHT structures). Although these experiments have been proven successful (Kulow *et al.*, 2020 ▸; Haboub *et al.*, 2014 ▸), this involved a significant loss of solid angle, with the open area reducing to at best 25%. We wanted to achieve the theoretical maximum open area of about 50%, so needed a supported structure. A thin layer of silicon nitride is ideally suited for such a support, and this is what we implemented. The optics were fabricated using the LIGA technique (Guckel *et al.*, 1991 ▸), at the X-ray lithography facility at CAMD, in Baton Rouge, Louisiana, USA. We prepared lithography masks with all of the patterns listed above, plus other objects which might be of interest (single pinholes, slits and more). Silicon wafers were coated with a 1 µm-thick layer of silicon nitride. One side was patterned with squares matched to the array sizes, and the nitride etched away in those locations. An orientation-dependent silicon etch removed the silicon to leave a thin nitride membrane window which is X-ray transparent. On the other side of the wafer, a seed layer of gold was deposited, followed by a deep lithography in a thick photoresist. Following development of the resist, gold was electroplated into the cavities remaining, forming the thick gold aperture structures. Finally the photoresist was removed and the seed layer etched away, leaving free-standing structures. Fig. 2 ▸ shows the finished wafer, and Fig. 3 ▸ shows electron micrographs of one of the structures thus formed.

## 3. Experiments   

### 3.1. Point source   

**Figure 4:**
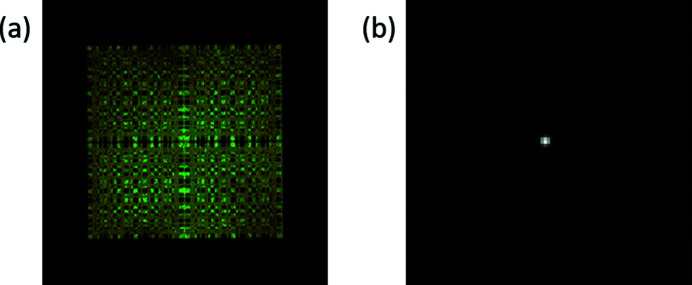
(*a*) Image of an XRG point source through an order 73 MURA on a 256 × 256 pixels Medipix detector. (*b*) Retrieved point source from the shadow of the XRG source through the order 73 MURA on the Medipix detector using total variation least-squares deconvolution.

Our first experiments used a microfocus X-ray generator (XRG) which was capable of providing a 10 µm focal spot from a molybdenum anode tube. We used the order 73 MURA to image that focal spot onto a Medipix detector. This detector has a 256 × 256 array of photon-counting pixels on a 55 µm pitch. It does not provide energy resolution, but can accept a high photon flux. The resulting image is shown in Fig. 4 ▸(*a*). Since the source is essentially a point source, the recorded image is a smeared shadow of the mask. It was easy to perform the reconstruction using total variation regularized least-squares deconvolution (Chan *et al.*, 2011 ▸), and the result is shown in Fig. 4 ▸(*b*).

### 3.2. Extended source   

**Figure 5:**
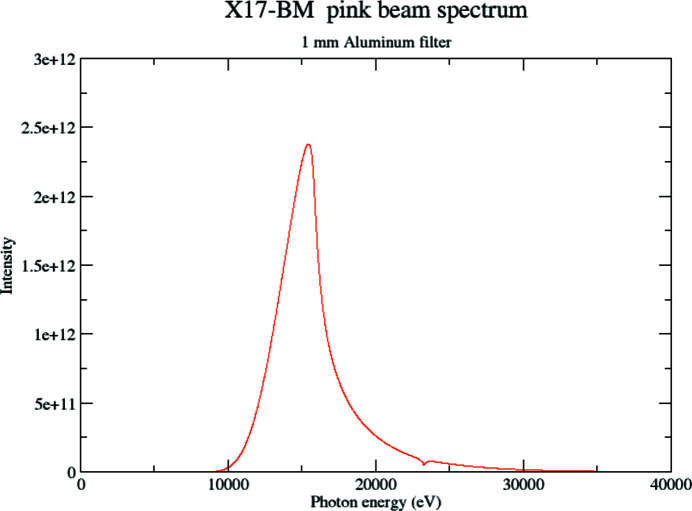
Calculated spectrum of the XFP pink beam with 1 mm-thick aluminium filter.

We used the XFP beamline at NSLS-II (Asuru *et al.*, 2019 ▸) to demonstrate that the image processing method would also work on extended and fluorescing samples. XFP can operate in a pink beam mode with a partially focused spot of roughly 2 mm × 2 mm. The optics of the beamline were optimized for low-energy photons, mainly below 20 keV, with the majority of the beam power below 10 keV. We added a 1 mm-thick aluminium filter upstream of our setup to reduce the thermal load this beam would place on any sample. Fig. 5 ▸ shows the resultant calculated spectrum.

**Figure 6:**
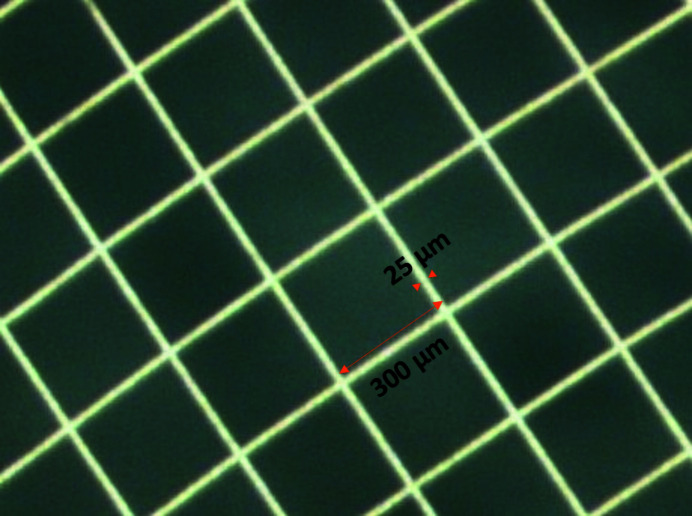
Optical micrograph of the gold wire mesh with 25 µm wires woven into a 300 µm-pitch square mesh.

We chose a gold wire mesh as our sample. The mesh is nominally 25 µm wires woven into a 300 µm-pitch square mesh (Fig. 6 ▸). It was placed across a rectangular frame, such that the incident beam only illuminated the mesh and air. The distance *d*_1_ between the object and the mask was 38 mm and the distance *d*_2_ between the mask and the detector was 571 mm. We used the same Medipix detector as the one described in Section 3.1. Given the geometry of the mask and the setup, the theoretical resolution *r* is calculated to be 14.3 µm as
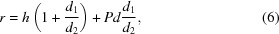
with *h* the size of the mask pinhole, 10 µm in our case, and *Pd* the size of the detector pixel which is 55 µm.

**Figure 7:**
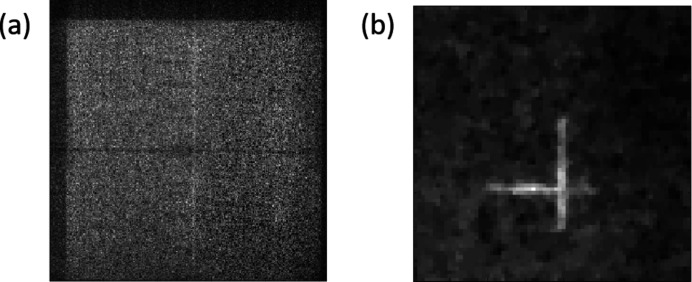
(*a*) Raw data on the 256 × 256 pixels Medipix detector from XFP fluorescence measurement of the gold wire mesh. The beam measured 200 µm × 200 µm and the mesh was oriented with its wires horizontal and vertical. (*b*) Retrieved image of the gold wire mesh obtained from using total variation least-squares deconvolution of the detector’s raw data.

The first image had the mesh oriented with its wires horizontal and vertical. It was illuminated with a small beam, approximately 200 µm × 200 µm. The raw data and reconstructed image are shown in Fig. 7 ▸. We observed approximately 22.4 µm wire width, which suggests that the actual resolution of the setup is less than 25 µm. The discrepancy in the wire width could easily come from uncertainty in the magnification which will be properly characterized in future measurements.

**Figure 8:**
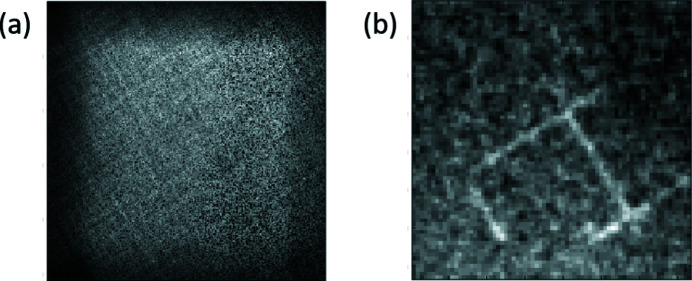
(*a*) Raw data on the 256 × 256 pixels Medipix detector from XFP fluorescence measurement of the gold wire mesh. The beam measured 600 µm × 400 µm and the mesh was oriented at 45°. (*b*) Retrieved image of the gold wire mesh obtained from using total variation least-squares deconvolution of the detector’s raw data.

We then oriented the mesh so that the wires were at roughly 45° to the horizontal, and enlarged the illuminating beam to 600 µm × 400 µm. The raw data and reconstruction are shown in Fig. 8 ▸. The contrast is reduced compared with the image with the smaller beam. We believe that this is due to the increased parasitic scattering from the larger beam, adding a uniform background to the data. This is probably a consequence of our decision to operate close to backscattering, rather than at 90° to the incident beam as is usually done for fluorescence measurements. Nevertheless, the image can be reconstructed successfully. Similarly to the point source, the images of the extended source [Figs. 7 ▸(*b*) and 8 ▸(*b*)] were reconstructed using total variation regularized least-squares deconvolution.

## 4. Conclusions   

Using the LIGA technique, we fabricated a MURA of order 73 with 10 µm holes and 50% open area, providing the maximum possible throughput for such a system. Using this optic we could reconstruct part of a gold wire mesh of 25 µm wires on a 300 µm pitch. This suggests that we reach a resolution better than 25 µm. The theoretical resolution for our setup is 14.3 µm. In our future work we will carefully characterize the magnification of the system and upgrade our image processing methods as suggested by Kulow *et al.* (2020 ▸). We will also design and fabricate apertures which match more closely the needs of the intended research, and develop a hybrid pixel detector in which each pixel is a spectrometer capable of handling a high rate and providing high-quality spectra.

## References

[bb3] Asuru, A., Farquhar, E. R., Sullivan, M., Abel, D., Toomey, J., Chance, M. R. & Bohon, J. (2019). *J. Synchrotron Rad.* **26**, 1388–1399. 10.1107/S1600577519003576 PMC6613119 31274468

[bb4] Chan, S. H., Khoshabeh, R., Gibson, K. B., Gill, P. E. & Nguyen, T. Q. (2011). *IEEE Trans. Image Process.* **20**, 3097–3111. 10.1109/TIP.2011.2158229 21632302

[bb7] Gottesman, S. R. & Fenimore, E. E. (1989). *Appl. Opt.* **28**, 4344–4352. 10.1364/AO.28.004344 20555874

[bb8] Guckel, K., Skrobis, K. J., Christenson, T. R., Klein, J., Han, S., Choi, B. & Lovell, E. G. (1991). *Proceedings of the IEEE International Conference on Micro Electro Mechanical Systems*, 30 January–2 February 1991, Nara, Japan, pp. 74–79.

[bb9] Haboub, A., MacDowell, A. A., Marchesini, S. & Parkinson, D. Y. (2014). *Rev. Sci. Instrum.* **85**, 063704. 10.1063/1.4882337 24985824

[bb10] Kulow, A., Buzanich, A. G., Reinholz, U., Streli, C. & Radtke, M. (2020). *J. Anal. At. Spectrom.* **35**, 347–356.

[bb11] Takeuchi, A., Terada, Y., Suzuki, Y., Uesugi, K. & Aoki, S. (2009). *J. Synchrotron Rad.* **16**, 616–621. 10.1107/S0909049509029598 19713634

[bb12] Takeuchi, A., Terada, Y., Uesugi, K. & Suzuki, Y. (2010). *Nucl. Instrum. Methods Phys. Res. A*, **616**, 261–265.

